# Intraspecific variations in cardamom (*Elettaria cardamomum* Maton): assessment of genomic diversity by flow cytometry, cytological studies and ISSR analysis

**DOI:** 10.1186/s40064-016-3226-x

**Published:** 2016-09-13

**Authors:** N. Anjali, K. M. Ganga, F. Nadiya, S. Shefeek, K. K. Sabu

**Affiliations:** Biotechnology and Bioinformatics Division, Jawaharlal Nehru Tropical Botanic Garden and Research Institute (JNTBGRI), Palode, Thiruvananthapuram, 695562 India

**Keywords:** Cardamom, Genome size, Genetic variation, Flow cytometry, 2C value, *Elettaria cardamomum* Maton, Zingiberace, ISSR, EST-SSR

## Abstract

**Background:**

The main goal of the work was to analyse intraspecific variation in *Elettaria cardamomum* Maton (cardamom) using genome size, cytological studies and molecular marker data. Nuclear DNA content and molecular marker details furnish data on genome size and genetic diversity respectively among the studied accessions and both complement each other for evolutionary and taxonomic studies.

**Results:**

The relative 2C genome size and total number of base pairs of cardamom was determined through flow cytometric analysis using propidium iodide staining. The nuclear DNA content was estimated in various sections of the species representing individuals from wild and cultivar genotypes following *Zea mays* L. CE-777 (2C = 5.43 pg) as internal reference standard. Chromosome number from growing root tip was examined following standard protocols. Twenty-six ISSR primers that generated polymorphic bands were used for genetic diversity analysis of the thirty accessions of cardamom. Estimated nuclear 2C DNA content ranged from 2.57 to 3.22 pg demonstrating 1.25-fold variation. The mean amount of 2C nuclear DNA of the cardamom was calculated as 2.87 pg which is equivalent of 2806 Mbp as the diploid genome size. The chromosome number was found to be 2n = 48. Among the thirty accessions of cardamom studied using ISSR markers, C53 (feral from Bonacaud) showed a very prominent level of genetic diversity and was lowest for C96 (Avinash-I, a released variety from Indian Institute of Spices Research, Kozhikode).

**Conclusion:**

These analyses revealed the existence of genetic variability within the studied cardamom accessions. The plant specimens also differed significantly in their genome size. However, the genetic variability parameters did not show any correlation with genome size.

## Background

*Elettaria cardamomum* Maton known as Queen of spices belongs to monocotyledonous family Zingiberaceae. This perennial rhizomatous plant is mainly confined to mid-elevations (600–1300 m a.s.l.) in the Western Ghats of South India (Ravindran and Madhusoodanan 2003). It is a shade loving plant and 40–50 % shade promote its vegetative growth and development (Kumar et al. 2015). Dried fruit of small cardamom (commonly called as cardamom) is widely used for culinary and medicinal purposes. It is mainly used as a flavouring agent in food preparations and for the treatment of gastrointestinal disorders. D-Limonene, one of the constituent of cardamom oil is reported to have tumour suppressing effect against colon, mammary, liver, lung, skin and stomach cancers in rodents (Samir et al. 2015). It has been stated in a US patent (US 20060147561 A1) that a poly herbal formulation including cardamom can be efficiently used for diabetic treatment (Nitasha Bhat et al. 2015).

Cardamom is indigenous to South India and Sri Lanka (Purseglove et al. 1981) and there is a strong belief that the species has been originated in the moist evergreen rainforests of the Western Ghats of South India (Kumar et al. 1997). According to reliable reports, cardamom cultivation began during 1803 in India and until then, cardamom pods were harvested from forests (Ravindran and Madhusoodanan 2003). This means that selection of cardamom with respect to better agronomic traits began around 210 years ago. Further, the cultivation was usually practised in forests after clearing all the undergrowth where cardamom grows naturally. These processes could have gradually eliminated wild cardamom as such and genetic variations there exist. Frequent cultivation of cardamom and targeted selection of peculiar morpho/ecotypes have resulted in the development of many location specific landraces. As in the case of other crop plants, wild cardamom plants (including disease escapes (Venugopal 1999) from abandoned plantations) may have genetic variability and possess alleles that can be effectively used for crop improvement programmes.

Genetic diversity is the basis for development of elite varieties with desirable characteristics. Genetic diversity analysis can be performed using morphological, biochemical and molecular markers (Govindaraj et al. 2015). The biological diversity information which is important for efficacious conservation programmes have become refined by the usage of molecular techniques (Oliveira-Miranda et al. 2013). Molecular markers offer consistent results despite the prevailing environmental circumstances. Among the widely used markers, inter simple sequence repeat (ISSR) marker is a PCR based molecular marker in which a DNA region situated between two similar microsatellite motifs aligned in opposite directions got amplified (Mohammad et al. 2015). So the primers used in this case are microsatellite portions which are tandem repeats of di- to penta-nucleotides. Knowledge on DNA sequence of study organism is not needed for ISSR marker study and can be undertaken for any plant species (Prakashkumar et al. 2015).

ISSR polymorphism has been widely used all over the world to characterize plant genetic variations. It is reported that Iranian *Hypericum perforatum* L. wild population shows genetic diversity at the intraspecific level estimated using ISSR markers (Mohammad et al. 2015). ISSR profiling revealed 87 % polymorphism in nine varieties of *Ginkgo biloba* (Zhiqiang et al. 2014). RAPD markers unravelled more information on genetic variability than ISSR markers in *Justicia adhatoda* L., *Caldesia grandis* and *Dalbergia sissoo* (Amit et al. 2014; Chen et al. 2006; Arif et al. 2009). High level of polymorphism was obtained for seven populations of *Lemna gibba* using five ISSR primers (Aziza et al. 2015). Remarkable level of polymorphism was detected among thirteen accessions of the medicinal plant *Rauvolfia serpentina* L. with fifteen ISSR primers (Padmesh et al. 2012).

Microsatellites occur in all plant genomes are studied widely for analyzing the genetic variations. They are abundant in non-coding genomic regions, but also detected in coding regions through studies using EST-SSRs (Ranade et al. 2014). For example, in EST databases, an EST-SSR occurs every 14 kb in *Arabidopsis* (Cardle et al. 2000) and every 19 kb in rice (Temnykh et al. 2001). In contrast to the genomic SSRs, EST-SSRs represent functional markers and changes in EST-SSRs length can cause a phenotypic effect (Li et al. 2004). The significance of EST-SSRs as a molecular tool in genetic studies is well known (Ellis and Burke 2007) and demonstrated in population studies and analysis of genetic diversity in *Populus* (Xinye et al. 2009); in hybrid selection in *Citrus* (Rao et al. 2008); and also in genetic mapping in *Pinus* (Echt et al. 2011) to mention a few. Furthermore, unlike the genomic SSRs, EST-SSRs are easily transferable across species (Kalia et al. 2011), therefore allowing studying polymorphism and genetic diversity in related species (Ellis and Burke 2007). Recently, it has been reported that microsatellites or simple sequence repeats from expressed sequence tags (EST-SSRs) of curcuma shows considerable genetic variation in cardamom (Anjali et al. 2015).

The plant cell nucleus consists of most of the hereditary material and has always been a subject of intensive studies. Nuclear DNA C-value is the amount of DNA incorporated within a haploid nucleus and is expressed in picograms (1 pg DNA = 0.978 × 10^9^ bp) (Dolezel et al. 2003). 2C-value specifies the amount of DNA contained within two copies of unreplicated genome in a nucleus which is in the G1 phase of the cell cycle (Dolezel and Bartos 2005). The First plant genome size determination was done in *Lilium longiflorum* in the year 1951 (Pellicer and Leitch 2014). Measurement of nuclear DNA amount has paved the way for uncovering broad range of difference in genome size in various organisms (Dolezel et al. 2007). No correlation exists between the genome size and organismal complexity as the eukaryotic genomes consist of large quantities of non-protein coding DNA. Genome size of more than 15,000 species of animals, plants, fungi and protists has been estimated so far (Elliott and Gregory 2015). Kew plant DNA C-value database is the depository for genome size data of plants (http://data.kew.org/cvalues/) and the commonly noticed genome size in Kew database is 500 Mb (Michael 2014). Genome size in Angiosperms ranges from 0.129 pg in *Genlisea margaretae* to 304.46 pg in *Paris japonica* (Chen et al. 2014). The important element which determines the genome size is the number of repetitive sequences (Sakurako et al. 1997). Genome size is related to change in periodic biological phenomena of plants, responsiveness to frost, ecological fitting in response to availability of water and organism, tissue, cellular and sub cellular level characters like nuclear and cell volumes, mitotic and meiotic cycles span and seed weight (Suda et al. 2007). Genome size knowledge is required for designing gene cloning, small RNA and whole genome sequencing projects as their scale and cost are determined by genome size.

One of the main applications of flow cytometry is the estimation of genome size. The simplicity in sample preparation, fastness, precision, convenience and the potential to check large numbers of samples per day make flow cytometry an efficient method for estimating genome size in plants (Yue-ping et al. 2015). The first fluorescence based flow cytometer was developed by Wolfgang gohde in 1968. Flow cytometer aids in measurement and counting of particles in a constrained suspension and comprises of three components—fluidics, optics and electronics (Sergio 2008). When illuminated by an intense source of light, these particles absorb and scatter light. If the particles are tagged with any fluorescent dye, it emit fluorescent signals also. The fluorochromes tether stoichiometrically with nucleic acids so that number of probe molecules bound is comparable to the number of DNA molecules. The scattered light and fluorescent signals are perceived by dichroic mirrors, filters and photomultiplier tubes (Galbraith 2010). The software equipped with the instrument translates these signals into a graph in which fluorescent intensity is plotted against cell counts (Sergio 2006). The discovery of such an effortless and precised method for isolating nuclei from solid tissues becomes an innovation in flow cytometry of plant species (Loureiro et al. 2010). The nuclear DNA content of the reference standard used should not be too close or too distant from that of the unknown sample (Milene et al. 2011).

Nuclear DNA content and molecular marker details furnish data on genome size and genetic diversity among accessions respectively and both complement each other for evolutionary and taxonomic studies. In the present study, thirty accessions of cardamom were subjected to nuclear DNA content estimation by flow cytometry and genetic variability analysis using ISSR markers. To the best of our knowledge, this is a novel study using two types of techniques which complement each other in deciphering the genetic architecture of cardamom genome.

## Methods

### Plant materials

Thirty accessions of cardamom including wild collections (those from natural forests), landraces, feral and released varieties were used for genome size estimation and ISSR marker study. All the samples were obtained from the germplasm collection of authors’ Institute. They were originally collected from different parts of Western ghats (Table 1). A portable GPS (Garmin, New Delhi) was used to document the geographic location of accessions collected.

**Table 1:** Details of cardamom accessions used for genome size estimation and ISSR analysis

Accession no.	Type/name	Location	Latitude	Longitude
C11	Palakkudi (LR)	Anakkuzhy, Idukki	N 09°37′31.09″	E 77°06′07.08″
C16	Feral	Pandimotta	N 08°50′06.11″	E 77°11′03.00″
C17	Feral	Pandimotta	N 08°50′06.11″	E 77°11′03.0″
C26	Wild	Rosemala	N 8°54′49.45″	E 77°10′55.45″
C28	Feral	Bonacaud	N 8°41′33.09″	E 77°10′45.09″
C31	Feral	Bonecadu	N 08°41′37.03″	E 77°10′48.03″
C38	Feral	Cheenikala, Sankili	NA	NA
C39	Feral	Cheenikala, Sankili	NA	NA
C40	Feral	Peppara	N 8°37′11.98″	E 77°11′47.87″
C51	Kanniyelam (Feral)	Maniyaramkudi, Idukki	N 9°53′45.78″	E 76°56′08.28″
C52	Feral	Agasthyamala	N 8°37′03.39″	E 77°13′45.56″
C53	Feral	Bonacaud	N 8°41′52.01″	E 77°10′53.0″
C55	Palakkudi (LR)	Idukki	N 9°41′04.0″	E 77°11′08.0″
C61	ICRI 2 (RV)	Idukki	NA	NA
C62	ICRI 5 (RV)	Idukki	NA	NA
C63	Valli Green Gold (LR)	Idukki	NA	NA
C65	PV2 (RV)	Idukki	N 09°47′48.0″	E 77°09′30.0″
C68	Wild	ICRI, Idukki	NA	NA
C70	Feral	Kakki, Periyar	N 9°18′55.08″	E 77°8′34.08″
C74	Wild	Variyam, Ernakulam	N 10°12′57.01″	E 76°51′26.08″
C75	Wild	Therakkudy, Ernakulam	N 10°13′13.20″	E 76°47′03.07″
C76	Wild	Kulirukadu,A ryankavu	N 9°1′09.02″	E 77°6′08.00″
C77	Wild	Valiyathalappara, Aryankavu	N 9°1′59.01″	E 77°7′59.05″
C81	Feral	IISR IC-349337, Karnataka	NA	NA
C83	Feral	IISR IC-349396, Karnataka	NA	NA
C84	Feral	IISR IC-349399, Karnataka	NA	NA
C86	Feral	IISR IC-349436, Karnataka	NA	NA
C91	Feral	IISR IC 349459, Karnataka	NA	NA
C96	Avinash-1 (RV)	IISR, Karnataka	NA	NA
C97	Appangala-1 (RV)	IISR, Karnataka	NA	NA

*RV* released variety, *LR* landrace. Both RV and LR are treated as cultivars

*Solanum lycopersicum* L. Stupicke polni rane (2C DNA content is 1.96 pg), *Glycine max* Merr. Polanka (2C DNA content is 2.50 pg) and *Zea mays* L. CE-777 (2C DNA content is 5.43 pg) obtained from Laboratory of Molecular Cytogenetics and Cytometry, Institute of Experimental Botany, Sokolovska, Czech Republic were used as internal reference standards for genome size estimation study (Dolezel et al. 2007).

### Genome size determination using flow cytometry

Samples were prepared according to a standard two step procedure (Otto 1990). About 50 mg of young and fresh incompletely expanded leaf tissue of test sample and internal standard were co-chopped with a sharp razor blade in a plastic petridish containing 2 ml of ice-cold Otto I buffer (0.1 M citric acid, 0.5 % Tween 20). This was kept at −4 °C for 40 min for releasing nuclei from cells to buffer. The nuclear suspension was filtered through a 40 µm Nylon mesh and centrifuged at 150*g* for 5 min. Nuclei were gently resuspended in 100 µl of fresh Otto I buffer after removing the supernatant. 1 ml of Otto II buffer (0.4 M Na_2_HPO_4_·12H_2_O) supplemented with propidium iodide for absolute DNA content determination and RNase (both at a final concentration of 100 µg/ml) was added (Bures et al. 2003; Leong-Skornickova et al. 2007; Lysak et al. 2000; Palomino et al. 2003; Pecinka et al. 2006; Roux et al. 2003). All the sample preparation methods were done on ice. The fluorescence intensity of 5000 particles was recorded using a BD FACSAria III Cell Sorter (BD Biosciences, California, USA). *Solanum lycopersicum* L. Stupicke polni rane, *Glycine max* Merr. Polanka and *Zea mays* L. CE-777 were used as internal reference standards to determine the genome size of cardamom and *Z. mays* L. CE-777 was found to be appropriate reference standard there exist around a twofold variation between the mean peak intensity of the sample and the standard (Petr et al. 2012). Each sample was analysed twice for getting reliable results.

The number of nuclei and coefficient of variation (CV) for peaks of both the cardamom and maize were procured by gating using the BD FACs Diva software. Rectangular gates were drawn manually around the section which is of interest on the histogram of PI fluorescence-Area against PI fluorescence-width to discard the intervention of debris. The mean peak intensity of nuclei of cardamom and maize in the G1 phase of the cell cycle were compared and 2C DNA content of cardamom was estimated (Wang et al. 2015). The formula used to estimate the nuclear DNA content of cardamom (Gregory et al. 2015) was: 2C DNA nuclear content (pg) of cardamom = (sample peak mean/standard peak mean) × 2C DNA content of the reference standard (pg). One way ANOVA and *t* Test were used to check the significance of genome size variation among the accessions (n = 30) (Sheidai et al. 2014).

### Determination of chromosome count

The root tips of cardamom were collected around 10.30 am which is the peak hour of mitotic activity in cardamom plants. Somatic chromosomes from root tip cells were fixed in Carnoy’s fluid (ethanol and glacial acetic acid in the ratio 3:1). The root tips were pretreated in 0.002 M solution of 8-hydroxyquinoline for 2 h at 4 °C. The smear and squash preparations were stained in 2 % acetocarmine, and well spread chromosome preparations were photomicrographed using Leica DM 100 digital camera attached with Leica DM 2500 trinocular microscope (Ngamriabsakul 2004).

### ISSR analysis

DNA was isolated from young leaf tissue of the samples using DNeasy Plant Mini Kit (Qiagen, Germany) following manufacturer’s instructions. The concentration and quality of the isolated DNA were checked using Biophotometer (Eppendorf India Ltd), 0.8 % agarose gel electrophoresis and was diluted to a final concentration of 50 ng/µl. Polymerase chain reaction (PCR) was carried out on the plant samples each in 25 µl reaction consisting of 50 ng template DNA, 1× PCR buffer (Origin, Kerala), 200 μM of each of the four dNTPs, 15 pM primer and one unit of Taq DNA polymerase (Origin, Kerala). Thirty-six ISSR primers (IDT, New Delhi) were checked and evaluated for clarity, consistency and number of polymorphisms. Twenty-six primers which created clear bands and amplification profile were finally selected for the study (Table 2). Amplification was carried out with the following conditions: 2 min at 94 °C, followed by 35 cycles of denaturation for 30 s at 94 °C, annealing for 1 min, extension for 2 min at 72 °C, and for 7 min at 72 °C for the final extension using Agilent Sure Cycler (Agilent Technologies, Malaysia). The amplified products were resolved with the aid of 1.4 % agarose gel electrophoresis in 1× TBE buffer and visualized using Safe View™ Classic (Applied Biological Materials, Canada) in gel documentation system (UVP, UK). 1 kb or 100 bp DNA ladder (Origin, Kerala) was loaded in the gel as the size marker.

**Table 2:** Details of ISSR primers used to analyze genetic diversity in 30 cardamom accessions

Sl. no.	ISSR primer	Sequence	Ta (˚C)
1	S807	5′-TATATATATATATATAC-3′	50.0
2	S809	5′-AGAGAGAGAGAGAGAGC-3′	52.0
3	S811	5′-GAGAGAGAGAGAGAGAT-3′	50.4
4	S813	5′-GAGAGAGAGAGAGAGAC-3′	52.0
5	S817	5′-CACACACACACACACAT-3′	50.0
6	S820	5′-CACACACACACACACAA-3′	50.0
7	S824	5′-TCTCTCTCTCTCTCTCC-3′	52.0
8	S826	5′-TCTCTCTCTCTCTCTCG-3′	52.0
9	S829	5′-ACACACACACACACC-3′	52.0
10	S834	5′-TGTGTGTGTGTGTGC-3′	52.0
11	S835	5′-AGAGAGAGAGAGAGAGYT-3′	50.0
12	S836	5′-AGAGAGAGAGAGAGAGYC-3′	50.0
13	S842	5′-GAGAGAGAGAGAGAGAYC-3′	53.7
14	S844	5′-CTCTCTCTCTCTCTCTRA-3′	50.0
15	S845	5′-CTCTCTCTCTCTCTCTRC-3′	53.0
16	S849	5′-CACACACACACACACARG-3′	53.7
17	S850	5′-GTGTGTGTGTGTGTGTCA-3′	53.7
18	S851	5′-GTGTGTGTGTGTGTGTTC-3′	55.8
19	S855	5′-TCTCTCTCTCTCTCTCRG-3′	51.0
20	S857	5′-ACACACACACACACACY-3′	53.0
21	S860	5′-ACACACACACACACACTG-3′	53.0
22	S864	5′-TGTGTGTGTGTGTGA-3′	47.0
23	S868	5′-CTCCTCCTCCTCCTCCTC-3′	47.0
24	S872	5′-GAAGAAGAAGAAGAAGA-3′	40.0
25	S880	5′-CTTCACTTCACTTCA-3′	51.0
26	S895	5′-GGAGAGGAGAGGAGA-3′	51.0

The bands obtained with each primer were scored as diallelic characters: 1 denotes present and 0 denotes absent (Lamyai et al. 2014; Mark et al. 2015). The amplified products extends in size from 200 to 2000 bp. A binary qualitative data matrix was formed and estimated the Observed number of alleles (*na*), Effective number of alleles (*ne*), Nei’s gene diversity (*h*), Shannon’s Information index (*I*) and number of polymorphic loci (*p*) using the software POPGENE ver 1.31 (Yeh et al. 1999; Amit et al. 2014).

In an attempt to get a deeper understanding of the relationship between genetic variability and genome size, 2C-values of the accessions which are common in the present study and that of our previous research (Anjali et al. 2015) were subjected to correlation analysis with ISSR and EST-SSR based genetic variability parameters such as number of alleles per loci and polymorphic alleles.

## Results

### 2C nuclear DNA content estimation

**Fig. 1:**
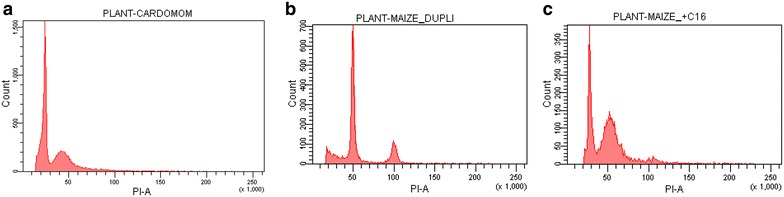
Representative flow cytometric histograms (**a**) cardamom (sample) alone (**b**) *Zea mays* (internal reference standard) alone and (**c**) documenting both cardamom and *Z. mays*

Flow cytometric study generated histograms with good resolution peaks (Fig. 1). Nuclear DNA content of thirty accessions of cardamom were estimated (Table 3). 2C DNA amount shows low variation among the studied accessions. Maximum genome size was 3.22 pg shown in accession C61 (ICRI-2, a released variety) and minimum was found in C39 (a wild accession from Cheenikkala, Sankhili) as 2.57 pg. It shows a difference of about 1.25 times. ANOVA and *t* Test analysis did not find out significant differences in nuclear DNA content among the 30 accessions analysed as wild, feral, and cultivar groups. Mean 2C nuclear DNA content of all the thirty accessions of cardamom was calculated as 2.87 pg and the mean genome size is 2.81 × 10^9^ bp. This is the first report on genome size of *E. cardamomum*.

**Table 3:** Genome size of the studied accessions categorized into various groups

Acc. no.	Type	2C DNA (pg)	Genome size (bp)
C11	LR	2.82	2.76 × 10^9^
C51	Feral	2.71	2.65 × 10^9^
C55	LR	3.02	2.95 × 10^9^
C61	RV	3.22	3.15 × 10^9^
C62	RV	2.68	2.62 × 10^9^
C63	LR	2.93	2.87 × 10^9^
C65	RV	3.10	3.03 × 10^9^
C96	RV	3.07	3.00 × 10^9^
C97	RV	2.84	2.78 × 10^9^
C16	Feral	2.83	2.77 × 10^9^
C17	Feral	2.89	2.83 × 10^9^
C28	Feral	2.96	2.89 × 10^9^
C31	Feral	2.69	2.63 × 10^9^
C38	Feral	2.78	2.72 × 10^9^
C39	Feral	2.57	2.51 × 10^9^
C40	Feral	2.77	2.71 × 10^9^
C52	Feral	2.95	2.89 × 10^9^
C53	Feral	2.72	2.66 × 10^9^
C70	Feral	2.72	2.66 × 10^9^
C81	Feral	3.04	2.97 × 10^9^
C83	Feral	3.14	3.07 × 10^9^
C84	Feral	2.75	2.69 × 10^9^
C86	Feral	2.85	2.79 × 10^9^
C91	Feral	2.74	2.68 × 10^9^
C26	Wild	2.91	2.85 × 10^9^
C68	Wild	3.14	3.07 × 10^9^
C74	Wild	2.74	2.68 × 10^9^
C75	Wild	2.70	2.64 × 10^9^
C76	Wild	2.97	2.90 × 10^9^
C77	Wild	2.86	2.80 × 10^9^

*RV* released variety, *LR* landrace

### Chromosome count

The chromosome number was found to be 2n = 48 in the studied accessions by observing the mitotic metaphase stage. The experimental was repeated with different samples, but no variation in chromosome number was observed.

### Genetic diversity analysis using ISSR markers

Twenty-six primers that generated polymorphic bands were used for genetic diversity analysis of the thirty accessions of cardamom (Table 4). Twenty-six primers generated 97 bands and polymorphism was shown by 85 bands (87.63 %). The number of polymorphic bands obtained for each primer extends from one (S849) to seven (S845). Shannon’s Information Index (*I*) differ from 0.23 to 0.54 with an average of 0.37. Among the thirty accessions of cardamom studied, C53 (feral from Bonacaud) showed a very prominent level of genetic diversity (*h* = 0.38, *I* = 0.54, *P* = 80.77 %) and was lowest (*h* = 0.16, *I* = 0.23, *P* = 38.46 %) for C96 (Avinash-I, a released variety from IISR).

**Table 4:** Genetic diversity indices of the thirty cardamom accessions studied using twenty-six ISSR primers

Accessions	*na*	*ne*	*h*	*I*	*P* (%)
C40	1.65	1.50	0.27	0.40	65.38
C26	1.62	1.49	0.26	0.38	61.54
C75	1.65	1.54	0.29	0.41	65.38
C74	1.69	1.57	0.30	0.43	69.23
C68	1.73	1.49	0.28	0.41	73.08
C70	1.69	1.54	0.30	0.43	69.23
C51	1.77	1.61	0.33	0.47	76.92
C17	1.73	1.64	0.34	0.48	73.08
C53	1.81	1.73	0.38	0.53	80.77
C28	1.65	1.58	0.31	0.43	65.38
C52	1.65	1.45	0.26	0.37	65.38
C38	1.50	1.40	0.21	0.31	50.00
C11	1.58	1.48	0.26	0.36	57.69
C76	1.62	1.50	0.27	0.39	61.54
C77	1.77	1.61	0.33	0.48	76.92
C16	1.61	1.53	0.28	0.40	61.54
C63	1.65	1.52	0.28	0.40	65.38
C31	1.58	1.47	0.25	0.36	57.69
C55	1.62	1.52	0.28	0.39	61.54
C61	1.54	1.38	0.21	0.31	53.85
C62	1.58	1.39	0.22	0.33	57.69
C65	1.58	1.46	0.25	0.35	57.69
C39	1.46	1.33	0.19	0.27	46.15
C91	1.38	1.34	0.18	0.25	38.46
C97	1.42	1.35	0.19	0.27	42.31
C96	1.38	1.30	0.16	0.23	38.46
C81	1.58	1.48	0.25	0.36	57.69
C84	1.46	1.36	0.19	0.28	46.15
C83	1.54	1.44	0.23	0.33	53.85
C86	1.54	1.40	0.22	0.32	53.85
Mean	1.60	1.48	0.26	0.37	60.12
SD	0.11	0.10	0.05	0.07	10.85

*na* = observed number of alleles, *ne* = effective number of alleles, *h* = Nei’s gene diversity, I = Shannon’s information index, *P* (%) = percentage of polymorphic loci

### Correlation between genome size and genetic variability

**Fig. 2:**
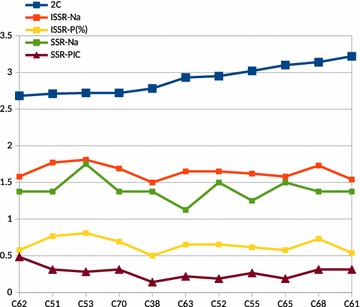
Relationship between 2C-value, ISSR and EST-SSR variability. *Na* stands for number of alleles per locus, *P* for percentage of polymorphic loci and *PIC* for Polymorphic Information Content

The analysis revealed no correlation between the genetic variability and genome size in the studied accessions (Fig. 2). Further, the thirty accessions were divided into four groups based on their wild/cultivar nature. The one-way ANOVA was estimated from these four groups, viz. feral [n = 16, mean (m) = 2.82, variance (v) = 0.0213], landrace (n = 3, m = 2.92, v = 0.0100), released varieties (n = 5, m = 2.98, v = 0.0474), and wild (n = 6, m = 2.87, v = 0.0257). Since if F(1.4992) < F(2.9752) crit, it was concluded that the four groups did not differ significantly from each other. Further *t* Tests were also performed to test each of the above pairs of the means. These tests also did not show any significant differences between any of the pairs.

## Discussion

It is obvious that different individuals of same species vary in genome composition due to various evolutionary reasons. Cardamom is one of the species domesticated since modern man started agricultural practices and with a strong desire to satisfy his appetite. Mentions of cardamom are available in old Indian literature written somewhere in 3000 BC (Ravindran and Madhusoodanan 2003). Cardamom cultivation has started in late nineteenth century. Since then, the natural populations of cardamom are slowly replaced by ‘selections’ which narrow down the genetic base and thus the evolutionary potential of cardamom. Some of the remaining of the natural populations of cardamom available in the pristine forest areas of Kerala were collected for this study and analysed along with other available genotypes (Table 1). The present study revealed that the distribution of the genetic diversity and differences in genome size in various accessions are highly randomized having no correlation with the type of the genotype (viz. wild, feral, released variety or cultivar).

Domestication generally leads to changes in many productive traits in crop species when compared to their wild progenitors (Colunga-Garcia et al. 1996; Colunga-Garcia and May-Pat 1997; Casas et al. 1999, 2007). In cardamom, domestication has increased the number of branches and the number of inflorescences per clump, which have resulted in a significant increase in the total number of flowers per clump. These features have obviously resulted in a significant increase in fruit yield in the cultivars (Kuriakose et al. 2008). Considerable variation occurs in cardamom, because it is cross-pollinated (Wardini and Thomas 1999). Studies show that there was no reproductive barrier between wild and cultivated cardamom populations (Kuriakose et al. 2008). Fruits with viable seeds were produced when the accessions used for the present study were subjected to manual cross pollination each other (Sabu and Shefeek, unpublished).

### Genome size estimation

Variation in nuclear DNA content was observed among the studied accessions of cardamom which ranged from 2.54 to 3.22 pg. However, this variation was not statistically significant as estimated using one-way ANOVA and *t* Test. This means that genome size estimates among the 30 accessions or among the various groups (wild, feral, released variety and cultivar) did not differ significantly from each other. According to various reports, intraspecific variation in nuclear DNA content of plants is a disputable subject in the scientific community. Very few studies are reported with remarkable level of intraspecific genome size variation (Bennett and Leitch 2005). As per some of the earlier studies on *Collinsia verna*, *Pisum sativum* and *Glycine max*, it was observed that there was a significant variation. But later Greilhuber (1998) came out with the conclusion that they were merely due to some technical issues. Based on some works which described the variation in genome size within species using authentic controls and standards, Murray (2005) explained that this intraspecific variation has some taxonomic relevance if the former could be related to morphological variation. About 1.19 times variation in genome size was reported for *Linum glaucum* populations in Iran and difference in nuclear DNA content was found to be associated with altitude and morphological characters (Seyed et al. 2015). Marek et al. (2009) reported a high level intraspecific variation in different populations of *Picris hieracioides*. Significant level of intraspecific variation was noted in five species of *Lathyrus* genus (Nandini et al. 1997). The contradiction of having low intraspecific variation and high interspecific variation in diploid Triticeae species proposed that emergence of genome size occurs just before, during or right after the process of speciation (Eilam et al. 2007). 2C nuclear DNA content analysis exhibited a 1.1 fold difference and thus a low level intraspecific variation in *Camellia sinesnsis* var. *assamica* (Huang et al. 2013). 1C nuclear DNA content of the populations of *Pinus nigra* was reported to have around 23 pg (Faruk et al. 2007). Sandra et al. (2015) described that DNA content shows only a small variation within *Miscanthus sinensis* cultivars. Considerable differences in DNA content was not observed among the five populations of *Pinus heldreichii* when a *t* Test was performed (Bogunic et al. 2003).

### Genome size in Zingiberaceae members

A search in the C-value database of RBG Kew using the term ‘Zingiberaceae’ resulted in 39 records belonging to 7 genus such as *Curcuma*, *Paracautleya*, *Hitchenia*, *Kaempferia*, *Stahlianthus*, *Alpinia* and *Zingiber*. Average 2C value of the family is 2.79 pg and the minimum and maximum values are 1.66 and 12.05 respectively. The mean 2C value of cardamom estimated in the present study was 2.87 pg which falls in comparable range of the family.

### Use of genome size estimation

In addition to chromosome numbers and ploidy levels, genome size has been estimated in many plants as it is an important attribute in biology and biodiversity (Bennett et al. 2000; Hanson et al. 2001) and pre-requisite for genome sequencing experiments. The co-processed samples with different genome sizes always gave two distinct peaks, which is the most convincing evidence for genuine differences in DNA content (Greilhuber 2005). Genome size variation has significant consequences at cellular, tissue and organismal levels and also influences phenological and ecological behaviour. Comparisons of genome size data within genus and species levels and probable phylogenetic relationships suggest that both increases and decreases of genome size have occurred in evolution (Wendel et al. 2002; Soltis et al. 2003).

### Ploidy level and genome size

The occurrence of different chromosome numbers (2n = 48, 52) in cardamom was reported in early cytological studies (Gregory 1936; Sharma and Bhattacharya 1959; Chakravarti 1948). Variations in chromosome numbers were observed in Mysore and Malabar varieties of cardamom indicted that aneuploidy as well as structural alterations in the chromosome have contributed to the varietal differentiation (Chandrasekhar and Sampathkumar 1986). Earlier workers have reported that cardamom is of amphidiploid origin from wild species (Peter et al. 2007). As ploidy level is well correlated with the mode of reproduction, detailed knowledge of genome size in particular species plays an important role in forming hypotheses about evolutionary potential of the species and evolutionary processes in the genus (Chrtek et al. 2007). The present study did not find any ploidy changes among the studied accessions as evident from observations based on cytology and the present flow cytometry analyses. In general, ploidy screening can be done through analysing a test sample with those of the same species having known ploidy using the same instrument settings. This would result in a histogram from which ploidy can be determined from the G1 peak position. The wild cardamom plant collected from Therakkudy, Ernakulam (C75) was screened for chromosome number by root tip squash technique and found to be tetraploid (2n = 4x = 48). While calculating the 2C DNA content of cardamom using maize as internal reference standard, the G1 peak of all the cardamom accessions were positioned around channel 26. So it can be concluded that all the 30 accessions of cardamom that were taken for this particular study are having tetraploid nature.

### Genome size and genetic variability

It is well cited that there is no correlation between the amount of DNA per cell and organismal advancement or genetic complexity (Sparrow et al. 1972; Price 1988) which has been historically termed the “C-value paradox” (Thomas 1971). Since the discovery of non-coding DNA and its impact on genome size variation [for example, retrotransposons have increased the size of the maize genome two- to five-fold since the divergence of maize and sorghum from a common ancestor about 16 million years ago (Sanmiguel and Bennetzen 1998)], “paradox” has been replaced by “enigma” in order to address the matter properly (Gregory 2002, 2004). It is now generally agreed that the C-value enigma is due to the differential amplification and proliferation of the repetitive sequences of the genome among organisms (Bennetzen 2000, 2002; Kidwell 2002; Hawkins et al. 2006).

Microsatellites are ubiquitous class of simple repetitive DNA sequences which are abundant in all eukaryotes analyzed and is thought to result from the mutational effects of replication slippage (Tautz and Schlötterer 1994). To find out the relation between genome size and microsatellite occurrence, the microsatellite frequency was analyzed in plant species with a 50-fold range in genome size that is mostly attributable to the recent amplification of repetitive DNA (San Miguel et al. 1998). It was found that the overall frequency of microsatellites was inversely related to genome size and to the proportion of repetitive DNA but remained constant in the transcribed portion of the genome among species (Morgante et al. 2002). This indicates that most microsatellites reside in regions pre-dating the recent genome expansion in many plants. The microsatellite frequency was higher in transcribed regions, especially in the untranslated portions, than in genomic DNA (Morgante et al. 2002). In a more recent study, it was found that EST-SSR density in the coding regions was not associated with genome size in selected genera of woody trees representing gymnosperms (17 species from seven genera) and angiosperms (40 species from eight genera) (Ranade et al. 2014).

In the present study, high genetic variation was observed among different accessions of cardamom using ISSR markers (for example, Shannon’s information index (Table 4) ranged from 0.23 to 0.53, around 130 % increase). Similarly, the variability in genome size was also clearly observed. However, there was no correlation between the increase in the genetic variability parameters and genome size in the studied accessions (Fig. 2) which is at par with a previous finding that EST-SSR density was not associated with genome size in neither angiosperms nor gymnosperms (Ranade et al. 2014).

## Conclusion

The present study focuses on intraspecific variation in cardamom with respect to genome size and ISSR data. Variability in genome size was found for different categories of cardamom like wild, feral and cultivar types. Genetic variability was analyzed among the thirty accessions using twenty-six ISSR primers. None of the genetic variability parameters showed a correlation with genome size. It could be concluded that the genetic variability exhibited by the different accessions of cardamom occurs due to minor insertion or deletion of nucleotide sequences. It might be reasonable to assume that retrotransposons that make up the majority of the plant genomes (San Miguel et al. 1998) could be attributable to the increased genome size in some of the cardamom genotypes.
